# Integrating Ambient In-Home Sensor Data and Electronic Health Record Data for the Prediction of Outcomes in Amyotrophic Lateral Sclerosis: Protocol for an Exploratory Feasibility Study

**DOI:** 10.2196/60437

**Published:** 2025-03-12

**Authors:** William E Janes, Noah Marchal, Xing Song, Mihail Popescu, Abu Saleh Mohammad Mosa, Juliana H Earwood, Vovanti Jones, Marjorie Skubic

**Affiliations:** 1 Department of Occupational Therapy College of Health Science University of Missouri Columbia, MO United States; 2 Institute for Data Science and Informatics University of Missouri Columbia, MO United States; 3 Department of Biomedical Informatics, Biostatistics, and Medical Epidemiology School of Medicine University of Missouri Columbia, MO United States; 4 Electrical Engineering and Computer Science Department College of Engineering University of Missouri Columbia, MO United States; 5 Physical Medicine and Rehabilitation School of Medicine University of Missouri Columbia, MO United States

**Keywords:** amyotrophic lateral sclerosis, machine learning, precision health, ALS, health monitoring, electronic health record, EHR, federated approach, in-home sensor data

## Abstract

**Background:**

Amyotrophic lateral sclerosis (ALS) leads to rapid physiological and functional decline before causing untimely death. Current best-practice approaches to interdisciplinary care are unable to provide adequate monitoring of patients’ health. Passive in-home sensor systems enable 24×7 health monitoring. Combining sensor data with outcomes extracted from the electronic health record (EHR) through a supervised machine learning algorithm may enable health care providers to predict and ultimately slow decline among people living with ALS.

**Objective:**

This study aims to describe a federated approach to assimilating sensor and EHR data in a machine learning algorithm to predict decline among people living with ALS.

**Methods:**

Sensor systems have been continuously deployed in the homes of 4 participants for up to 330 days. Sensors include bed, gait, and motion sensors. Sensor data are subjected to a multidimensional streaming clustering algorithm to detect changes in health status. Specific health outcomes are identified in the EHR and extracted via the REDCap (Research Electronic Data Capture; Vanderbilt University) Fast Healthcare Interoperability Resource directly into a secure database.

**Results:**

As of this writing (fall 2024), machine learning algorithms are currently in development to predict those health outcomes from sensor-detected changes in health status. This methodology paper presents preliminary results from one participant as a proof of concept. The participant experienced several notable changes in activity, fluctuations in heart rate and respiration rate, and reductions in gait speed. Data collection will continue through 2025 with a growing sample.

**Conclusions:**

The system described in this paper enables tracking the health status of people living with ALS at unprecedented levels of granularity. Combined with tightly integrated EHR data, we anticipate building predictive models that can identify opportunities for health care services before adverse events occur. We anticipate that this system will improve and extend the lives of people living with ALS.

**International Registered Report Identifier (IRRID):**

DERR1-10.2196/60437

## Introduction

Amyotrophic lateral sclerosis (ALS) is a rapidly progressing neurodegenerative disorder that usually leads to death within 3 years of symptom onset and within 2 years from diagnosis [1]. The sequence and rate of ALS progression can vary widely depending on the phenotype, age at onset, and diagnostic delay [2,3]. Pharmacological developments (eg, Riluzole and Radicava) can slow disease progression, but the result is always premature death, typically due to respiratory failure, pneumonia, or heart failure [4,5].

Treatment in a specialized, multidisciplinary ALS clinic is among the strongest predictors of quality of life and prolonged survival among people with ALS [6]. In the United States, these clinics take the form of ALS Association Certified Treatment Centers of Excellence, which, by definition, must include a multidisciplinary team with treatment standards based on the American Academy of Neurology Practice Parameters [6,7]. Even in this best-case treatment setting, though, weeks or months may pass between clinic visits. Rapid physiological and functional declines between visits mean that a person with ALS can be hospitalized or deceased before the care team is aware of their change in status. The time between clinic visits represents a critical gap in the multidisciplinary care of people with ALS. A reliable, noninvasive system for passively detecting physiological and functional decline between visits would give the multidisciplinary team unprecedented ability to predict outcomes and, more importantly, make near–real-time treatment recommendations to counteract disease progression, prolong independence, and maintain quality of life. Rates of respiratory decline and lower extremity functional decline, in particular, are reliable prognostic indicators for ALS survival time [1]. In addition to pharmacological options, targeted nonpharmacologic interventions can reduce perceived fatigue, improve manual dexterity, prevent falls, promote myriad cognitive improvements, and preserve the overall quality of life among those living with ALS [8]. These interventions, enabled by early detection of decline, are most effective when delivered in the context of multidisciplinary programs, resulting in longer survival and higher quality of life [6,9].

To monitor the decline, members of the research team have developed a passive, in-home, sensor-based system for monitoring physiological biomarkers and functional status through a combination of hydraulic bed sensors, motion sensors, and privacy-preserving depth sensors [10]. The system can reliably capture pulse, respiration rate, bed restlessness, room activity, gait speed, stride length, and falls. It has been deployed in more than 300 senior housing units and private homes throughout the midwestern United States since 2005. The system was developed and tested in two phases with support from the National Institutes of Health (National Institute of Nursing Research 1R21NR011197-01, Rantz, principal investigator). The first phase was retrospective [11-13], reviewing 3 years of significant health events from previous study participants. Parameters deemed important for an aging-in-place population include increasing or decreasing bed restlessness, pulse, respiration, time in bed, increasing bathroom activity, and decreasing general activity and time away from home [10,14-16]. Previous iterations of the system have been described extensively elsewhere [17,18]. The current iteration is a closed-source implementation purchased from Foresite Healthcare and maintained by the research team.

Our previous work makes clear that it is possible to detect and track very early signs of health changes using passive in-home sensing. In some cases, the onset of clinical declines may be detected before patients are aware of changes [18-20]. The system captures a variety of important biomarkers and functional data, including the key variables for predicting decline among people with ALS. The purpose of this work is to adapt the existing sensor system to predict physiological and functional decline in people living with ALS. This approach relies on a myriad of multimodal data, including robust sensor signals and structured electronic health record (EHR) data from distinct platforms. Here, we describe a necessarily federated approach to combining these distinct data types into a single dataset for the generation of the prediction algorithm. We hypothesize that this approach is feasible.

## Methods

### Design

The goal of the parent study is to adapt the existing sensor-based alert system to facilitate early detection of physiological and functional declines among people living with ALS. To this end, the study considers three hypotheses: (1) the enhanced sensor system is feasible for collecting biometric data and health outcomes from people living with ALS; (2) enhanced sensor system data, processed through an unsupervised machine learning approach, may enable early detection of health status changes among people living with ALS; and (3) changes in health status detected by the multidimensional streaming clustering approach can predict adverse health outcomes, including pneumonia, hospitalization, and death among people living with ALS.

This paper specifically addresses the methodology for hypothesis 1, testing the feasibility of the federated collection, storage, and analysis of data from the sensor system and EHR. Data collection is scheduled to continue through December 2025, and interim findings are reported in the *Results* section below. Full results of this process will be reported elsewhere upon completion of the study.

### Recruitment

Participants are recruited from the ALS Association Certified Treatment Center of Excellence at University of Missouri Health Care. Inclusion criteria are age 18 years or older, a clinical diagnosis of ALS by a qualified neurologist as documented in the EHR, home zip code within 100 miles of the clinic, and either the presence of a live-in caregiver (eg, spouse or adult child) or a Montreal Cognitive Assessment (MoCA) score >22. Recruiting coordinators are allowed to omit scoring individual MoCA items if, in their clinical judgment, response difficulties are due to speech or motor deficits resulting from ALS, rather than cognitive impairment. ALS genotype and phenotype, enrollment in clinical trials, and any other treatments are not considered inclusion or exclusion criteria.

### Sensor System

We use three types of in-home sensors: hydraulic bed mats, thermal depth sensors, and motion tags, to monitor participant health. The sensor systems function effectively regardless of lighting conditions, providing a noninvasive, passive way to monitor participant health. The bed sensor is installed beneath the participant’s mattress, where signals are best captured during rest. It uses a hydraulic pressure transducer to gather composite ballistocardiogram signals, which are deconvolved into components of sleep restlessness, respiration rate, and pulse. Ballistocardiogram signals are a mechanical measure of blood flow produced by cardiac activity, analogous to electrocardiogram signal patterns. The composite signals are partitioned into their respective components using signal-filtering algorithms. The ballistocardiogram component is subjected to a sixth-order bandpass filter with a 0.7-10 Hz cutoff. The respiration component is subjected to a sixth-order low-pass filter with a 0.7 Hz cutoff. Instances of heightened bed restlessness appear in the signal data as periods marked by higher amplitude and increased noise.

A wall-mounted thermal depth sensor generates a series of depth images, where each pixel corresponds to a coordinate measurement within the scene [17]. The depth images contain 3D point-cloud coordinates of the participant during walks. Validated algorithms extract gait parameters from these depth images. Gait parameters for stride time, stride length, walking speed, and participant height are derived from the point cloud silhouettes. To overcome the limitations posed by the sensor's field of view, which can be potentially occluded by furniture or other objects, a centroid-based gait parameter estimation technique has been developed. This approach permits the collection of additional gait parameters, such as gait bounce, trunk sway, asymmetry, and entropy across all 3 axes (X, Y, and Z), as well as the XY diagonal with partial leg occlusion during stride. In addition to gait parameters, a standardized Timed Up and Go fall risk assessment score is computed from the average in-home walking speed, as captured by the depth sensor [17]. In the event of a detected fall, the depth sensor performs a dual function: sending an immediate alert to designated contacts and generating a short video clip of the fall event for subsequent investigation or diagnosis.

Passive infrared motion sensors are deployed using the ZigBee (Connectivity Standards Alliance) protocol, a high-level radio communication protocol designed for low-power wireless data transmission. The motion sensors are installed in key locations such as the bathroom, bedroom, living room, kitchen, and front door to capture room-level activity. Abnormal activity in these spaces, based on the time of day or night, may suggest potential health issues, such as a urinary tract infection or the onset of cognitive decline. As the motion sensors detect movement in infrared light, they are effective at identifying activity regardless of lighting conditions. In addition to motion counts, we calculate room activity density, represented as the number of motion events within a unit of time for a respective sensor, providing an overall level of activity for each room.

The system has been tested in a multiresidential environment and can handle the presence of visitors, distinguishing them from residents based on gait patterns [18]. In a commitment to maintain privacy and comply with HIPAA (Health Insurance Portability and Accountability Act) standards, all data collected from these sensors are anonymized and time stamped with a study-specific participant identifier, which is used to deterministically link with sensor data. The data are stored in a secure AWS instance.

### EHR Data Integration

We leveraged the REDCap (Research Electronic Data Capture; Vanderbilt University) Fast Healthcare Interoperability Resource (FHIR) interface to extract participants’ most recent clinical information directly from the backend Oracle Health EHRs Database system of University of Missouri Health [21-23]. The REDCap FHIR interface enables interoperability with EHR systems, allowing real-time data extraction. By leveraging the FHIR interface, we can extract relevant data elements, such as laboratory results, vital signs, and clinical assessments, to populate the registry with up-to-date information. To ensure data accuracy and completeness, we map the extracted EHR data to the corresponding REDCap data fields using standardized terminologies, such as *ICD-9-CM* (*International Classification of Diseases, Ninth Revision, Clinical Modification*), *ICD-10* (*International Statistical Classification of Diseases and Related Health Problems 10th Revision*), LOINC (Logical Observation Identifiers Names and Codes), SNOMED CT (Systematized Medical Nomenclature for Medicine–Clinical Terminology), and RxNorm. This mapping process facilitates seamless data integration and enables efficient data retrieval for further analysis. Once the EHR data are extracted and mapped to REDCap data fields, the data are stored in the REDCap database. REDCap provides a secure and user-friendly platform for managing research data, allowing researchers to more easily access clinical information on people with ALS. However, the current FHIR interface does not support real-time extraction of unstructured notes, which were manually extracted and uploaded into the same REDCap project, along with an additional outcome ascertainment form for collecting monthly evaluations of the ALS Functional Rating Scale-Revised (ALSFRS-R). All EHR data collected into REDCap are anonymized and time stamped, with a study-specific participant identifier created to deterministically link with sensor data.

### Data Management Plan

The extracted EHR data from REDCap and sensor data are loaded into a single study-designated cloud storage via Secure Shell File Transfer Protocol or Transport Layer Security 1.2 Protocol, hosted on a HIPAA-compliant, cloud-based data enclave. Data are integrated and analyzed via the self-service “Analytic Workbench,” hosted within the same data enclave. Uninterrupted monitoring of accesses and activities occurs on the study database following established best practices that have been implemented at the system level. Researchers’ access details are required to be reviewed on an annual basis according to current data use protocols. Access to the study database is restricted to the study period, plus an additional 5 years after the end of the study period, to facilitate subsequent requests to validate and reuse the database for future analyses and retrospective projects [24].

### Data Analysis

#### Sensor Feature Engineering

We will test the preliminary efficacy of the expanded sensor platform for detecting changes in health status among people living with ALS. The Center for Eldercare and Rehabilitation Technology has previously developed a multidimensional streaming clustering algorithm that can simultaneously monitor all of the inputs of the existing sensor platform and detect cumulative deviations from expected patterns. The multidimensional streaming clustering approach applies principal components analysis and t-distributed stochastic neighbor embedding to identify the “normal” relationships between multiple biometric variables. The change in each variable over time can be treated as a vector, and those vectors can effectively be summed to produce a single vector leading to a single point in feature space. The point represents the patient’s cumulative health status for the day, and the vector indicates day-to-day changes in that overall health status. When tracked over time, these changes reveal a cluster of expected behavior. The cluster is defined not only by its central mean value but also by the degree of typicality of the values. Typicality is an indicator of the consistency within the cluster, comparable with SD. Importantly, those clusters remain relatively stable and identifiable within a given period. Potential alterations in function can then be identified by retrospectively tracing the daily point trajectory as it approaches or leaves the cluster boundary. This deviation from typicality is often a warning sign of health decline caused by injury or illness. Outliers indicate potential moments of acute health decline, triggering a warning to health care staff. This same approach will be leveraged using expanded sensor platform data (including input from the Garmin 245 wearable sensor) to generate and monitor clusters of baseline data for people with ALS.

To establish the preliminary efficacy of the system, we will follow the methods demonstrated by Wu et al [25]. We will retrospectively compare outlier points to any adverse health outcome indicators from the EHR to explore whether detected outliers occurred in the period immediately preceding adverse health outcomes. This process begins with an exploratory visual analysis conducted by the Center for Eldercare and Rehabilitation Technology and Clinical Teams. We can then bin outliers into true and false positives and bin adverse health events into caught and missed events, establishing the sensitivity and specificity of the outliers as alerts to health status change. These results will achieve two important goals. First, they will answer the preliminary efficacy question, providing our first indication of whether this system can potentially detect health status changes in people living with ALS. We will also investigate the associations between the outliers and ALSFRS-R score changes and irregularities.

#### Early Detection Modeling

We will develop multiple state-of-the-art machine learning models (including but not limited to regularized logistic regression, least absolute shrinkage and selection operator, support vector machine [26], random forest [27], gradient boosting, and deep neural networks [28]) to achieve optimal performance for predicting future ALSFRS-R scores. Machine learning approaches broadly involve three phases: training, validation, and testing. In the training phase of a supervised learning approach, investigators define the relevant predictors (eg, biometric sensor data and outlier warnings) and outcomes (eg, ALSFRS-R score change, EHR indicators of pneumonia, hospitalization, and death) of interest. The system feeds the predictors and outcomes into a recurrent neural network first to extract sequential features, and then each of the identified algorithms creates a predictive model that can subsequently predict outcomes in novel datasets. We will perform the training phase on the first 12 months of retrospective sensor and EHR data. When the training and validation phases involve continuous collection of data from a single or growing dataset, the holdout method suggests a 2:1 ratio of training to validation data. Therefore, we will complete the validation phase with the final 6 months of prospective data collection. In the validation phase, inputs (eg, biometric data, gait, and sleep) are fed into the model and predicted outputs (eg, adverse health outcomes) are compared to real-world outcomes (eg, EHR outcome indicators of pneumonia, hospitalization, and death) [29]. Validation tests are then conducted for both traditional sensitivity and specificity, as well as for overfitting of the model. The validation phase allows us to fine-tune the relative weights of each factor in the model, improving its predictive validity. The optimal predictive model will be selected based on testing accuracy.

### Ethical Considerations

This study was approved by the University of Missouri Institutional Review Board (project number 2084262) and the U.S. Army Medical Research and Development Command (USMRDC) Office of Human Research Oversight (log number E03062.1a). All participants provided informed consent. Sensor data are deidentified on the device before transmission to the REDCap database. EHR data are deidentified in the REDCap FHIR process before transmission to the REDCap database. Participants are not compensated.

## Results

### User Statistics

**Figure 1:**
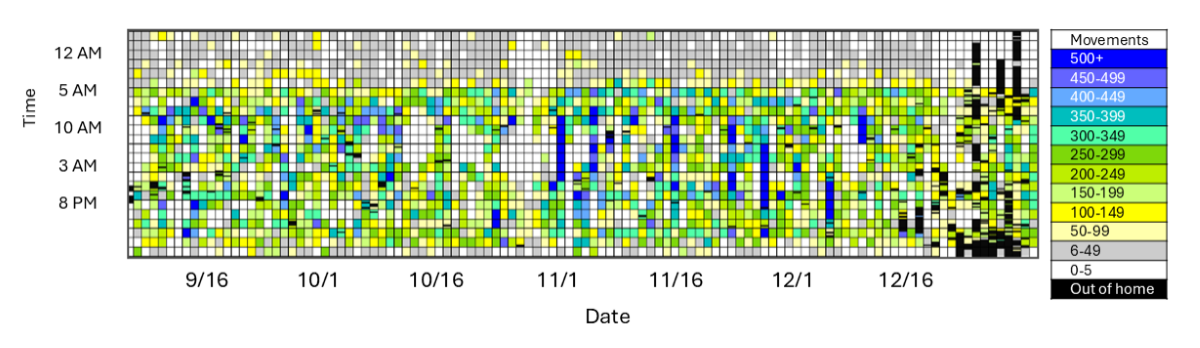
Whole home movement data density by hour from September 11 to December 29, 2023.

**Figure 2:**
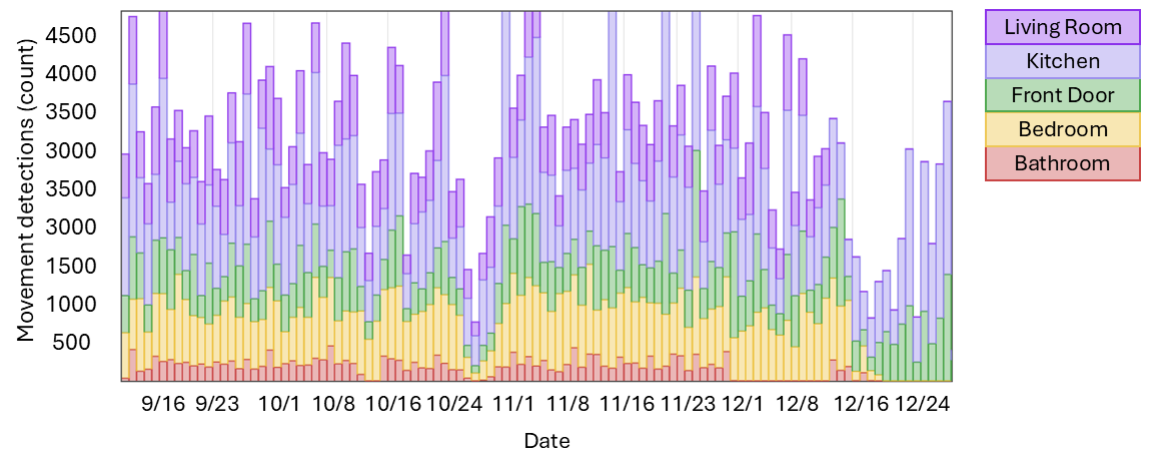
Per-room movement detections over 24-hour periods from September 11 to December 29, 2023. Red: bathroom; yellow: bedroom; green: front door; lavender: kitchen; purple: living room.

**Figure 3:**
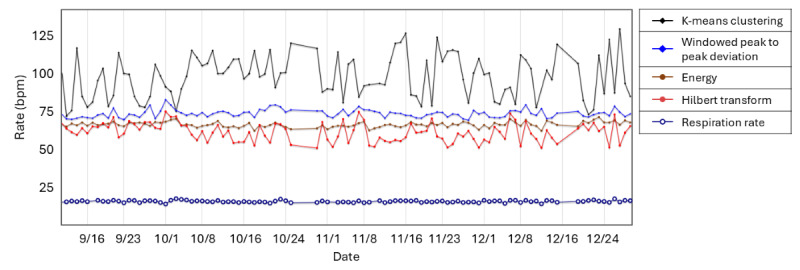
Heart rate and respiration rate by day from September 11 to December 29, 2023. Brown: energy; red: Hilbert transform; black: K-means clustering; blue: windowed peak-to-peak deviation; purple: respiration rate.

**Figure 4:**
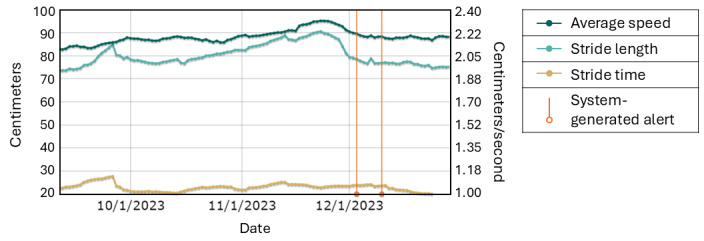
Gait data by date from September 11 to December 29, 2023. Light green: stride length (cm); dark green: average speed (cm/s); yellow: stride time (s); orange: system-generated gait alerts.

A total of 4 participants have been recruited (Table 1). None of the 4 participants required accommodations to complete the MoCA. Preliminary data from participant 1 are summarized in the preliminary analysis below (Figures 1-4). Specific dates of diagnosis and enrollment are not provided, as they could be combined with general geographic information to effectively deidentify participants with a rare condition. Sensor feature engineering and early detection modeling, based on all 4 participants, will be analyzed and reported in 2025.

Examples of monitoring results are shown in Figures 1-4, all scaled to the identical period from September 11 to December 29, 2023.

Figure 1 depicts motion density in the home, with the most motion detected during a prolonged family visit in November and considerable time spent out of the home around the December holidays (black boxes).

Figure 2 illustrates the number of movements detected in different rooms of the home, featuring spikes in time spent in bed (tall yellow lines) and confirming several nights spent outside the home in December (absence of yellow and red lines).

Figure 3 displays in-bed respiration rate (bottom purple plot) and four techniques for estimating pulse rate: energy [30] in brown, Hilbert transform [31] in red, k-means clustering [32] in black, and windowed peak-to-peak deviation [33] in blue.

Figure 4 depicts, from top to bottom, average gait speed, stride length, and stride time. Notably, reductions in average gait speed are indicated by orange lines on December 3 and 10, 2023. These represent system-generated alerts. The December 3, 2023, alert reads:

Walking Speed Decrease: The average walking speed of 91.4 cm/s observed during the current 7-day period ending on 12/03/2023 is 3.4 cm/s (3.6%) lower than the average walking speed of 94.8 cm/s observed during the 7-day baseline period which ended on 11/26/2023.

**Table 1:** Participant characteristics.

Characteristics	Participant			
	1	2	3	4
				
Age (years)	62	70	55	45
Sex	Male	Male	Male	Male
Race	White	White	White	White
Days since Dx^a^ at enrollment, n	617	41	24	56
Initial ALSFRS-R^b^ composite score	31	20	38	27
Deceased	No	Yes	No	No
Days of data collected, n	330	48	86	59

^a^Dx: diagnosis.

^b^ALSFRS-R: Amyotrophic Lateral Sclerosis Functional Rating Scale-Revised.

### Evaluation Outcomes

**Figure 5:**
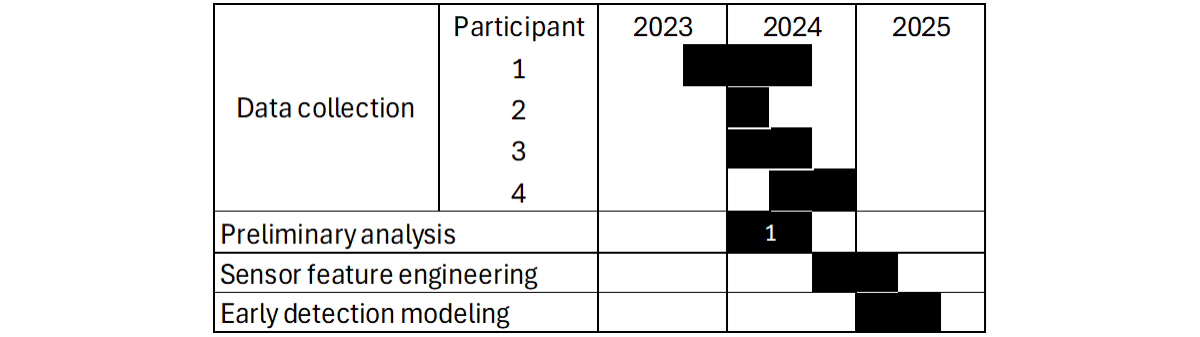
Timeline. “1” indicates the phase of work presented in this paper.

As of this writing (fall 2024), sensor feature engineering is underway, and early detection modeling will be reported upon completion. The timeline of this work-in-progress is provided in Figure 5.

## Discussion

### Preliminary Results

This paper describes a federated approach to the collection of in-home sensor data and the extraction of EHR data to create predictive models of health status change in people living with ALS. This approach is feasible for collecting both biometric data and health outcomes from people living with ALS. The sensors are nonintrusive and privacy preserving by design, making them acceptable to participants. Similarly, the FHIR process is nonintrusive and ensures timely, secure extraction of records for machine learning purposes.

In the next phase of this work, we will process the data using both supervised and unsupervised machine learning approaches to detect health status changes and predict adverse health outcomes, including pneumonia, hospitalization, and death among people living with ALS. We anticipate that the results generated by this protocol will inform subsequent clinical trials for the prediction of ALS progression, providing guidance for interventions and their optimal timing.

### Impact

This work represents a novel application of a remote sensor monitoring platform that has been previously validated in other settings and populations, such as stroke and independent living. This approach has tremendous potential in ALS, where progression is often rapid and, until now, unpredictable. It allows us to capture around-the-clock health state parameters, including during the critical late stages of decline. The system may provide important insight into physiological changes in late-stage ALS and suggest new intervention strategies to slow the rate of decline throughout the course of the disease.

### Limitations

We anticipate several challenges during the course of this study. The sensor data are limited to physiological measures, gait parameters, and household-level motion activity, which may not adequately capture the full spectrum of deficits observed in ALS and are reported through clinical evaluations such as the ALSFRS-R. Recruitment has been lower than projected, which could reduce our ability to develop a reliable predictive model. Even if the sample size meets projections, there remains concern regarding whether the sample will be representative of the larger population of people living with ALS due to the geographic limitations of our single-site study. Our sampling frame results in a sample that is disproportionately White and non-Hispanic. This is an inherently criterion-referenced, single-subject approach, where each patient’s biometric indicators are evaluated only in terms of deviation from their own established historical norms. In contrast, hypothesis 3 will involve creating a predictive model based on historical data. A model built from a homogeneous sample may overfit the characteristics of that sample. Overfitting occurs when relatively few outcome events are represented in the data, thus overweighting their contribution to the model. As a result, the model becomes tuned to detect cases that match the original training phase cases but cannot adapt to novel cases that differ from the training cases. As such, our initial model may not perform as well with people with ALS from non-White and Hispanic backgrounds, including Black people with ALS, who are known to receive later diagnoses and have faster rates of decline [34]. Our validation phase will recognize the limited diversity available in our sample. Importantly, we will design the initial model to evolve with additional data.

### Future Work

The project described in this paper will conclude in 2025. The authors have secured additional funding from the ALS Association (24-AT-722) to develop models to predict ALSFRS-R scores from sensor data and develop sensor-derived clinical alerts. That multisite project will provide a more diverse sampling frame to improve representation in the model. Future plans include clinical trials to establish the efficacy and effectiveness of this approach for delaying adverse outcomes.

### Conclusions

This study will provide important new insights into the progression of ALS at a previously impossible level of granularity. The federated system described in this paper will facilitate the development of predictive algorithms for illness, falls, hospitalization, and death. This approach has the potential to transform the clinical assessment workflow through the implementation of decision-support predictive analytics, enabling more proactive and personalized care strategies. By integrating sensor-informed care for clinicians and caregivers, we hope that this work may lay the foundation for clinical trials to intervene ahead of adverse outcomes with the goal of extending and improving the quality of life of people living with ALS.

## Abbreviations

ALS: amyotrophic lateral sclerosis
ALSFRS-R: Amyotrophic Lateral Sclerosis Functional Rating Scale-Revised
EHR: electronic health record
FHIR: Fast Healthcare Interoperability Resource
HIPAA: Health Insurance Portability and Accountability Act
ICD-9-CM: *International Classification of Diseases, Ninth Revision, Clinical Modification*
ICD-10: *International Statistical Classification of Diseases and Related Health Problems, 10th Revision*
LOINC: Logical Observation Identifiers Names and Codes
MoCA: Montreal Cognitive Assessment
REDCap: Research Electronic Data Capture
SNOMED CT: Systematized Medical Nomenclature for Medicine–Clinical Terminology

## Data Availability

The datasets generated and analyzed during this study are available from the corresponding author on reasonable request. This paper adheres to the Guidelines and Checklist for the Reporting on Digital Health Implementations (iCHECK-DH) checklist, a copy of which is available from the corresponding author upon reasonable request [35].
